# Survival Tactics and Strategies of Methamphetamine-Using HIV-Positive Men Who Have Sex with Men in San Diego

**DOI:** 10.1371/journal.pone.0139239

**Published:** 2015-09-30

**Authors:** Theodore K. Gideonse

**Affiliations:** Center for World Health, Division of Infectious Diseases, Department of Medicine, David Geffen School of Medicine, University of California, Los Angeles, Los Angeles, California, United States of America; Emory University's RSPH, UNITED STATES

## Abstract

In this article, two ways that HIV-positive drug users survive under the supervision of law enforcement agencies, community health organizations, and social welfare offices are differentiated. First, *strategies* are long-ranging and often carefully planned, and they involve conscious utilization and manipulation of bureaucratic processes. Second, *tactics* are short-ranging and often haphazard, and they are used to survive on daily or weekly bases, with entrenched problems and structural solutions avoided or ignored. Data from three years of ethnographic fieldwork with 14 methamphetamine-using HIV-positive men who have sex with men in San Diego, California is used to expand upon these two categories, explaining the different, often ineffectual, ways these men accessed care, services, shelter, drugs, and companionship. This article also examines the policy implications of taking in consideration these different kinds of survival methods, arguing for intensive client-specific interventions when working with long-term addicts with multiple health problems.

## Introduction

Methamphetamine use and HIV disease comprise a syndemic in American gay communities, particularly so in San Diego, where both meth and HIV have been endemic for almost four decades [1–3]. San Diego’s large gay community nevertheless has an 18% HIV prevalence rate [4], and meth is a problem in San Diego across multiple demographics: A quarter century ago, a spokesman for the Drug Enforcement Administration declared San Diego “the meth capital of the world,” and the only reason it cannot still claim that title is that other cities have since developed similarly high rates of meth use. In 2010 in San Diego 30% of people seeking treatment for substance abuse have problems with meth, while the national average is just 5% [5], and in 2013, 36% of men and 41% of women arrested in San Diego County tested positive for meth [6].

At the turn of the century, methamphetamine became the most popular drug, after alcohol and marijuana, among men who had sex with men (MSM) in the Western United States, and it still is [7,8]. In a 2008 survey, 50% of MSM in California had done meth in their lifetime, compared to 5% of the general population, and 15% of MSM 18–50 claiming to have used the drug in the last six months, compared to 1% in the general population [9,10]. Along with numerous physical sequelae associated with meth use, including major neurological and cardiovascular problems, MSM who take meth are much more likely to exhibit sexual behavior that puts them at high risk for contracting HIV [11–15].

Because meth use is associated with petty and violent crime as well as the spread of HIV and other STDs, and because of a moral panic inspired by these problems [16,17], public health and law enforcement agencies have responded with substantial, but sometimes ineffective efforts. These range from increased and aggressive policing [18] to restrictions of precursor ingredients for the manufacture of meth [19] to advertising campaigns devoted to demonizing the use or the users of meth. The collection of government and non-government organizations that focus either partly or exclusively on meth use and its sequelae like HIV, homelessness, addiction, and crime is extensive and difficult to navigate, with or without an addiction to a powerful stimulant. But in order to survive, addicts must develop methods to deal with these various institutions, laws, regulations, and the people who enact them.

Understanding the strategic (long-ranging, proactive) and tactical (short-ranging, reactive) methods that people like HIV-positive methamphetamine-using MSM use to survive can lead to better policies and more effective interventions. This article examines how several men operate their lives in concert with and in opposition to the various attempts to turn them into sober, economically self-sufficient citizens, exemplifying the problems inherent in developing and implementing interventions and policies focused on people whose behaviors, identities, and lives are often enacted in opposition to things like interventions and policies. While assertive community treatment (ACT), or intensive case management, has been shown effective with similar populations, it must be fully funded, the case managers trained well, and implemented with a clear and comprehensive understanding of individual history and psychology.

## Methods

### Study Design

This article is based on data collected between 2009 and 2012 for an anthropological study of methamphetamine-using HIV-positive men who have sex with men in San Diego, California, focused specifically on how they constructed their identities while under the supervision of multiple government and non-government institutions. The issue of identity is addressed in other publications [20,21], while this analysis is centered on how the men in the study subsisted on daily basis. In addition to participant observation among agencies and organizations focused on prevention and care of HIV/AIDS or methamphetamine addiction, the centerpiece of the research was person-centered ethnographic interviews with 14 HIV-positive MSM who use crystal meth. Thirteen men sat for full cycles of interviews, meeting with me between five and seven times for an hour. (One of the 14 only came twice; he died before follow-up interviews could be held.) Person-centered ethnography involves multiple unstructured interviews with informants over several months in addition to observation of the informants in their day-to-day activities, in this case mostly through shared meals and home visits. The method’s focus “is on the individual and on how the individual’s psychology and subjective experience both shape, and are shaped by, social and cultural processes” [22]. The goal of using this method in this study was to see how the study subjects crafted their identities and developed behaviors in reaction to both the cultural discourses and governmental actions surrounding HIV/AIDS, methamphetamine, and substance abuse in general.

For the HIV-positive methamphetamine addicts, managing daily life and surviving under government focus and disapproval is a great struggle. By using multiple, in-depth interviews and observations, I hoped to understand how the men in my sample were oriented to this cultural milieu [23] and how they survived their quotidian existence. I paid particularly close attention to how their individual classes, ethnicities, and other subject positions affected their narratives and identities. Similarly, these positions played roles in how the men in the sample experienced and expressed their emotions. I asked about their emotions, and I also tracked the emotional discourses in their narratives. During participant observation with the subset, I recorded how emotions were communicated and expressed [24–26], and I paid keen attention to how these experiences and self-concepts were physically expressed and mediated [27,28].

I recruited my study participants through referrals from healthcare providers, case managers, and addiction counselors, and other study participants. The sample was stratified and reflected the racial diversity of previous studies on MSM who use crystal meth in Southern California [7,13,15,29]. Seven were white, three African-American, two Hispanic, one Native American, and one was a Pacific Islander (see Table 1). However, because of where my referrals were originating, many of the men who joined my sample were in case management, in a rehabilitation program, or recently in recovery. While there are several different kinds of treatment programs for methamphetamine abusers, the men I interviewed rarely were treated with anything other than a 12-step program; this is largely because participating in Crystal Meth Anonymous, Narcotics Anonymous, and Alcoholics Anonymous is free. Four of the men were for a time part of a group that used the Getting Off program, a gay-specific cognitive behavioral therapy (GCBT) intervention [30], but that program folded after funding ended. Three others were briefly treated in residential programs using adaptations of the Matrix model [31], an evidenced-based program combining social support, education, and individual counseling. These three all left early or were unable to stay longer than a month. Nine of the 14 had been incarcerated at least once. Only one of the 14 was fully employed, and only one was a full-time student. The rest were either living off public assistance, through petty crime, or were homeless or some combination of all of these. So, while the socioeconomic statuses of their childhoods were diverse, most of my sample were similarly economically strapped. I paid each of these informants $15 in cash for each hour of interview time, and I conducted the vast majority of the interviews in a one-room office in downtown San Diego. All interviews in the office were audio recorded and supplemented with written notes; field notes were written during out-of-office shared meals and home visits.

**Table 1:** 

Name (Pseudonym)	Place of birth	Race/Ethnicity	Age at first interview	Age tested HIV+	Age of first meth use	Ever homeless	Ever incarcerated
*Adam*	Coldwater, MI	White	31	31	22	Yes	Yes
*Brandon*	Poway, CA	White	22	20	12	No	Yes
*Charles*	Placerville, CA	Native American	41	17	15	Yes	Yes
*Darrell*	National City, CA	African-American	36	34	17	No	No
*Eric* *	Johnson City, TN	White	46	22	25	No	No
*Glenn*	Burbank, CA	White	42	31	34	No	No
*Jonathan*	Santa Monica, CA	White	50	30	25	No	No
*Jorge*	Guadalajara, Mexico	Hispanic	47	28	42	Yes	No
*Matthew*	San Francisco, CA	White	32	26	12	No	Yes
*Max*	The Philippines	Pacific Islander	38	37	34	Yes	Yes
*Richard*	Stockton, CA	Hispanic	49	34	42	No	Yes
*Sam*	Berkeley, CA	White	43	23	13	Yes	Yes
*Walter*	Los Angeles, CA	African-American	49	36	49	No	Yes
*William*	San Francisco, CA	African-American	43	23	17	Yes	Yes

* Eric sat for two one-hour interviews in 2010 and did not return. He died the next summer in an altercation with police.

### Data Analysis and Theoretical Framework

Data were analyzed using grounded theory and Atlas.ti, with thematic domains emerging during the fieldwork process and codes and coding following interview transcription. The main aim of the study was to address issues of identity construction and emotional expression, and these analyses are discussed in other publications. This paper is based on the data that emerged in discussions with study participants of their day-to-say lives and how they subsisted, in both material and existential ways, while being tracked, policed, and the focus of various behavior modification programs. These issues were salient to the study’s main aim of identity construction because of the study’s governing theoretical framework in which identity is constructed and solidified through continual practice [32]. The praxis of survival then is a key element in the identity construction of the men in this study.

Different methods of survival can help to understand different kinds of identities. To help differentiate between the weaker and stronger methods of survival that the men in my study utilized, I turned to de Certeau’s concepts of “tactics” and “strategies” that he used to describe uses of space in *The Practice of Everyday Life*. The latter refers to the “calculation (or manipulation) of power relations” that can be made (or done) when something with “will and power” can be specifically placed and can “serve as the base from which relations with an *exteriority* composed of targets or threats (customers or competitors, enemies, the country surrounding the city, objectives and objects of research, etc.) can be managed.” Thus, the HIV clinic, the case manager’s office building, the police station, and the jail are places of strategy. Tactics, however, do not have the strength of place nor the power over space that strategies do. “The place of a tactic is the space of the other,” de Certeau writes. “Thus it must play on and with a terrain imposed on it and organized by the law of a foreign power. It operates in isolated actions, blow by blow. It must vigilantly make use of the cracks that particular conjunctions open in the surveillance of the proprietary powers… In short, a tactic is an art of the weak” [33]. Tactics are everything from sobriety checkpoints and profiling to HIV prevention outreach events in bars and anti-drug billboards.

Transferring de Certeau’s concept to human action and individual agency, we can see strategies as methods used with an awareness of power relations, both macro and micro-physics of power. They are long-ranging, usually carefully honed, and they involve consciousness of political economy, class, race, and general hierarchies and processes. The methods can be resistant to the broader strategies of institutional power, but they do not need to be; they can utilize, incorporate, or manipulate the other strategies. They involve the prudent spending of cultural and social capital. They involve an awareness of the agent’s place within the larger space; it is not just the feel for the game, but the knowledge of the position being played. While strategies are proactive, tactics are reactive. Tactics involve the awareness of only the micro-physics of power, with larger processes as mystifying as gravity or the weather. Tactics are methods used to survive day to day, maybe week to week, but they fail when applied to entrenched, structural problems. Robert Desjarlais used de Certeau’s concepts of strategies and tactics to analyze the rhetorical methods of residents of a drug treatment program [34]; in this paper I use the concepts to analyze series of actions and collections of behaviors over longer periods of time and in larger milieus.

### Ethics Statement

The Human Subject Research Program and the Institutional Review Board of the University of California, San Diego approved Project 091551, “The Effects of Cultural Conceptions of Health on HIV + MSM Who Use Crystal Meth,” on approved on October 9, 2009. Participants signed written consent forms, and these forms and the study protocol were approved by UCSD’s IRB. The names and some identifying characteristics of all study participants have been changed to protect their privacy.

## Results

Two different methods emerged from the interview and observational data regarding how the men survived, how they accessed scarce resources, and how they operated under the supervision or suspicion of law enforcement and public health agencies. Six of the 14 had been homeless at some point, while nine of the 14 had been incarcerated, and only two were employed during the study; all struggled in their daily lives to stay sober, to find drugs, to avoid punishments, to find food, to find shelter, to maintain adherence to HIV and other medications, or some combination therein. Contrary to my initial hypothesis, drugs were much more of a daily concern to the men than HIV was. High-quality HIV treatment in San Diego was free because of the federal Ryan White CARE Act and was described as eaily obtained, but that was not case for addiction treatment, which was rarely cheap and much less successful. The men rarely complained about complications of being HIV-positive, but did so constantly about addiction.

Much of their methods were tactical. For example, Adam (White, 31) would get money for drugs and food through petty theft and odd jobs, as well as through a network of men who gave him money or drugs in exchange for sex. It was chaotic process, and he often failed; when he was arrested for shoplifting food from Walmart, the police found methamphetamine on him, sending him to jail. Jonathan (White, 50) seemed to be in a constant state of anxiety about where his money and housing would come from, and his methods to get both worked briefly, but they never long-term solutions. He slept on couches of friends and acquaintances until they kicked him out, and he was able to borrow money but his friends until they realized he would never pay them back.

Strategies are more difficult to develop and follow through on, but by definition they have a longer-lasting positive impact. Glenn (White, 43), for example, relapsed many times during his long battle with methamphetamine addiction, but he talked about a vision of himself as a present and doting father and grandfather, and he slowly structured his life to make that more likely. He left the temptations of the gay meth scene in San Diego for Nebraska, where he was able to enroll in college, use his children as a support system, and more easily follow the tenets of the 12-step meetings he attended. While he has relapsed in Nebraska, he has done so less often.

To illuminate the complex context of these survival methods in their development and utilization, I describe in the following narrative vignettes three different methods (strategies and tactics) that have had long-term, if different degrees of, success. Two represent polar opposite strategies, and one a series of tactics that have worked for decades but may not continue to. These three are chosen because they show particularly clearly how the methods are tied to the men’s identities and self-understandings, justifying an approach to treating men like the study participants as individual cases needing individual plans of intervention

### Max

Max could have been put on a poster proclaiming the success of the 12-step program. It helped him stay sober, but he was also keenly aware that the program provided him with strategies for accessing resources. A slight, broad-smiling Filipino-American referred to me by the facilitator of a support group for HIV-positive gay men addicted to meth, when he began the study, he had been living in a group home for recovering addicts. He had stopped using meth three months before, when he tested positive for HIV. This event led him to get off the streets, where he had been living since leaving his boyfriend, who was also a meth addict.

Max was the only member of my sample who did not relapse during my fieldwork. He was not the only one whose narratives were structured by 12-step language and ideology, but he was the only one who seemed to be following steps almost exactly as they were laid out. Over the several months he was interviewed, he would gleefully provide updates on his progress, not only on which step he was working on, but also on his health: his increased weight, his lowered T-cell count and viral load. He also explained his various successes at the group home: He was running support groups, manning the front desk, supervising the other residents’ work, both domestic and recovery.

Max explained how the daily routine–morning meditation, taking medications, life skills training, group discussions, doctor’s appointments, chores, meals, and so on–helped him feel like he was part of a family. While other informants complained about how their day was over-scheduled by recovery programs, Max loved it. He told me he was always on time to appointments, went to his Narcotics Anonymous meetings like clockwork, met with his sponsor twice a week. He liked his sponsor, because “he is like a strict teacher.” He embraced the limits, and this made him particularly popular among the staff. He was quickly running the groups and supervising other residents. “I learned a lot about myself,” he said of the classes and 12-step meeting he attended daily. “I learned how to say no. I learned more responsibility to myself than to anyone else. I learned a lot running the groups. I’m very proud of myself. The big thing is grow up: I was spoiled, a mama’s boy. I learned a lot about being mature. If I follow the rules and use the tools, I will get what I need.”

Max was an enthusiastic model recovering addict. Accepting the hail of the recovering addict, and then aggressively defining himself within that identity, practicing its rituals, and preaching its benefits was strategic. He described that by following all of the rules and guidelines of his drug counselors, he gained more privileges in the home, a higher status among his fellow residents, and access to the so-called “normal” world of employment, leisure time activities, consumerism, and dating: “If I follow the rules and use the tools [of recovery], I will get what I need.” At the graduation ceremony from the recovery program, and when he was called onto the stage by the program’s director, the staff and the residents cheered more loudly for him than anyone else. When he accepted his diploma, he thanked the staff and his friends for helping him, but he singled out his “higher power” for doing the most to help him achieve sobriety and entrance back into the normal world. By the end of my fieldwork, he eventually made his way through barber school, was making money, had moved to an apartment, and had a large sober social network.

### William

William was an active meth user who was more self-controlled and self-possessed than any of the study subjects, even those who were sober during the study. This performance was part of a set strategies he developed to be able to get high and still stay out of jail, forced treatment, or other troubles. He was the only active user in my sample who never appeared to be high when he came to meeting, presenting the same measured affect that the sober research subjects did. He was always wearing clean clothes, his beard was trimmed, and he was always exactly on time. When it was noted that he seemed sober despite telling me that he was used meth regularly, he said the he made sure to be sober or near-sober for the hours that he had to interact with doctors, case workers, nurses, court officials, and researchers. When it was suggested that maybe he was not as addicted as other meth addicts, some of whom injected the drug, he said, “I’m a meth addict. I just maintain very well.”

William was living on disability, which he was able to get because of a lengthy AIDS-related hospital stay that he experienced in the late 1990s before he started taking the cocktail of anti-retroviral medications. Like Max, William had figured out a way to survive as an object of focus by institutions focused on HIV and substance abuse prevention. However, William does not survive because he was willing to be interpolated and assimilated by the recovery programs, as Max was. William’s method is creative and strategic resistance to the efforts to control his behavior and his subjectivity. He lived on SSI, had his own apartment greatly subsidized by HOPWA (Housing and Urban Development’s Housing Opportunities for Persons with AIDS), had a computer on which he watched TV and played internet-based games, and saw his HIV doctor at a community clinic regularly. He was well fed and had a good viral load and T-cell counts. He was also able to smoke meth regularly and have sex with men and women, some of whom were sex workers. While he was certainly not thriving according to those who see being a “productive member of society” as the base-line for morality, William was more than surviving. He had figured out how to stay out of jail, to maintain his government benefits, to keep his viral load low and his T cells high, to maintain his addiction, and to stay, at least based on the eight interviews I had with him, relatively cheerful, despite having a life that was the opposite of his childhood dream of being an upper middle class mechanical engineer.

William learned how to “maintain,” to present himself as sober, respectable, harmless, and not worthy of suspicion through a lifetime of being profiled as the opposite. He grew up in Oakland during the 1970s, which was both the height of the city’s African-American population’s political radicalism and the city’s heroin and gang-based crime wave. His elementary schools were violent, and he had to learn how to fight to survive. He also developed a consciousness about race and class influenced by the radical politics of Oakland at the time. While he was learning to avoid the police by becoming as close to invisible as possible, he was also learning how to make invisible other parts of his life from his family, neighborhood, and community. He had discovered that men would pay him for sex, but he knew no one else could know: “At the same time, I’m still going to school, and I’m doing very well in school. Now I’m leading a double life. I’ve got a girlfriend, I’m kicking ass in school, I’m on ROTC drill team, I’m running track, I’m wrestling… And I was going to the bookstores [in the red light district], and I was making money.” He ended up at San Diego State University’s prestigious engineering program on scholarship.

While William was lucky to have survived so long, while much of his survival came from his decision, he said, to “get smarter.” Part of that is figuring out how to handle the police and the policing. While San Diego in the 2000s was not policed with the same sort of excessive verve as Oakland in the 1970s and 1980s, William still feels the pressure of police harassment. But he learned how to, if not avoid the policing, how to manage it, either through modulating his interpersonal interactions or utilizing his knowledge of the law.

I can’t really beat the system. I can blend in with the system. If there’s a cop who’s harassing you, call him ‘Sir.’ The ends justify the means. The end is to walk away from his punk ass… If you show them the respect they are so desperately seeking they’ll tend to let you go.

William knew that he had little chance of succeeding in a constitutional challenge to a stop-and-frisk in Downtown San Diego. So, it was best to avoid any contact: “If you dress right…if you’re going to use dope tonight, you dress like you’re doing to party [at the downtown bars]. You can blend in. Because they’re acting like a fool, too. But if you dress a certain way, you don’t got a shot in hell. That’s why I dress a certain way.”

He did not “maintain” simply to avoid being arrested and to make paid research interviews, but also because he needed to be his own advocate in his HIV treatment. When his concerns and his questions were dismissed by doctors because of his meth use, he taught himself how to talk to his doctors as an educated health consumer, leading his doctors, he said, to respect him more. He did his own research on supplements, refused some recommended drugs because of side effects he discovered online, and told me he talked back to doctors who did not respect him. He also figured out how to persuade HOPWA to move him from the apartment under martial law, and he figured out ways to keep his math tutoring money off the radar of the Social Security Administration. He often spoke of his methods as political, that he was fighting racial oppression and countering the injustice of the Drug War. His resistance to the anti-meth efforts and institutions was hypercognized, carefully considered, and successful. De Certeau referred to strategies as methods that were calculated, manipulated, and made with awareness of power relations. He was fully aware of both the tactics and strategies employed by the Drug War itself, and he countered them one by one. He was able to extract the resources offered to HIV-positive people without giving up the meth and the meth-related behavior that made others in similar situations the object of harassment and derision.

### Sam

Sam grew up in East County San Diego. He first used meth when he was 13 and then began what he called “heavy” use when he was 16. He dropped out of school at 14 and left home permanently at 17, moving to Hillcrest to live with friends. He realized at 18 that his blond surfer looks made him attractive to men who were willing to pay for sex; he often hustled them, stealing from them after sex. He also made money from stealing cars, and he went to prison for the first time for auto theft. He tested positive for HIV in 1991, when he was 23 and in prison for auto theft. He is pretty sure that he contracted HIV from one of his johns. But at the time, he says, “I wasn’t gay.” He married Cathy, another addict who hung out in Hillcrest, in 1991. She was also positive. They got divorced in 1996, and then remarried. She died of AIDS in his arms in 2000, the same year he became homeless. Sam lived in a homeless encampment in a leafy canyon between a bridge, a highway, and an on-ramp in central San Diego called Camelot. Sam lived there off and on for 13 years, moving out when he’s been in jail, in a recovery problem, staying with a friend, or when San Diego’s police decide that they need to clear the area.

That he has survived so long, especially with a very weak adherence to his HIV medications, is a marvel to him and, as he told me, to his various doctors and case managers. A large factor is probably biological. His HIV did not progress quickly nor become resistant to medication, and his body has managed to withstand the large amounts and different kinds of drugs he has been addicted to. “According to the doctor, I should have been dead a long time ago,” Sam told me. The other factor is that he has developed a network of friends and charitable acquaintances, some of whom are also homeless or nearly homeless addicts but many of whom are not, who assist him in the belief that he is actually going to get better, get off the streets, and become a productive member of society. In one of the letters he sent to me from jail, he convinced me that he was planning to go into a recovery program when he was released with a statement typical of those he presented to people in this network:

I'm 44 and I'm not really going nowhere. What do I have to lose [by going to] one more drug program? It's a way to get back on my feet and off the streets. I want to go back to work so bad. I love to drive and tow cars so possibly towing or delivery driver or something like that. At—least I would be doing something that I enjoy therefore chances are I would stick with it. I know that I'm worth more than living in that damn canyon in Hillcrest.

One day Sam called me six times and sent me a dozen text messages. He had texted that “big changes R hapN soon” and his voicemails mentioned that he was meeting a new case worker, that he was getting his act together. I had been teaching and then working at the syringe exchange, and I texted him back saying that I would call him the next day. After that, he called me two more times and texted five more times, finally asking, “Could u get meSome food?” When I didn’t respond immediately, he texted, “Pleas I meet to talk2nite.” Then five minutes later, “have Re: Have a gond nite I bum at mc donalds loV u.” He had “bummed” a hamburger from a friend after his requests from me had not been answered. In the three years I have known Sam, I have bought him several meals, bought him a pack of cigarettes, and twice given him $20 after a series of desperate phone calls and texts; I also paid him $15 for each hour I interviewed him for a total of $90. Periodically, he would bombard me with calls and texts when he needed something, whether it was a small amount of cash, credits for his phone card, or, once, Xanax to help him come down from meth during one of many times he tried to quit. I stopped assisting Sam in material ways after a couple years, and I felt terribly guilty about setting up boundaries between us. But I recognized a pattern in his requests for help; each of his requests came after or in the midst of promises and plans for recovery, for treatment, for going to back to work as a tow truck driver. One afternoon, I watched him call a half dozen different friends to ask for money, a bed, or a ride (it seemed to depend on whom he called) and couple each request with a promise to go to a recovery program tomorrow, see his doctor, or sign up for a job program.

Like Max, Sam knows how to converse in the mantras and clichés of 12-step programs. In my many conversations with him, he has said that he needs to take it one day at a time, that God could help him if he went to church and prayed more often, that he just wants to get clean so that he can work, and if he could work, he would have a reason to stay clean. He wants to be sober, he has said many, many times. But unlike Max, Sam has never been able to stay sober. Like William, he has figured out how to get what he needs–food, shelter, medicine–from “the system.” But unlike William, Sam has no consistent method and little control over his behavior. He complains about how hard it is to get a free bus pass or to get the requisite paperwork for ADAP completed and submitted. For an addict, these straightforward but burdensome tasks can seem Sisyphean. He told me, “It’s a fucking full time job.” And sometimes he just refuses to go to work. Depending on his enthusiasm and focus, sometimes he goes to a food bank and sometimes he shoplifts. He is aware that the police are watching him and he says he needs to walk a certain way so that they will not suspect that he is high. It never works, however; in his tentative, nervous fidgeting, he is immediately suspicious to police, and so he gets stopped, frisked, and arrested on a regular basis. He does not see any of his difficulty as the result of political economic forces, as William does. Sam blames all of his hardships on himself, his lack of strength, his failure to be disciplined and responsible.

He has internalized the prohibitionist, 12-step discourses of the anti-drug efforts, but because he cannot figure out how to become that clean, sober, and productive member of society, he can only use them in the future subjunctive tense in his arguments for why someone should help. In conversations with me, he has listed all of the things he will do tomorrow to find a bed in a recovery program before asking me for money for a phone card. He has used similar stories in order to get more needles from the needle exchange than policy would allow him to have. I have heard him call one friend and after another to ask for favors–a bed, a ride, some money–and use the possibility of his recovery as reason for why the favor is both needed and justified. This sounds manipulative, and it is, but Sam is not doing it cynically. He is not devious. His relationships are not built simply on economic reciprocity. His capacity for empathy and for caretaking is great. He helps many of the other homeless addicts with procuring food, drugs, and other kinds of help, and when he asks them or asks me or the various outreach workers how they are, he seems actually to want to know. Many of the qualities that make him empathic also, when under the influence of meth, can make him hyper-sensitive and paranoid, but they also lead him to be more likely to be helped by his friends and by those in the HIV/AIDS service organizations and substance abuse programs who know him best. For a host psychological and biological and structural reasons, becoming sober may forever be elusive for Sam. His tactic of developing relationships of assistance, sometimes mutual and sometimes simply emotional, may keep him alive indefitinely, but quality of that life will never be high.

## Discussion

Among this study’s informants, the likelihood of being tactical and the ability to be strategic seemed to be dependent on several factors. Most importantly were biological, with both drug addiction and HIV disease affecting cognitive abilities and physical health in ways that can make strategic decision making, performances, and relationships within these complex fields of power difficult. Mental health problems, either as a result of or a reason for drug abuse, can also make long-range planning challenging. Socio-economic factors were also significant; a better education, as William had, can improve the ability to navigate and manipulate bureaucracies and to understand the political and economic processes that sustain Drug War policies and restrict access to quality healthcare. This was the case with William, as discussed above, as well as with Richard (Hispanic, 49) and Eric (White, 46), the only two participants with college degrees. The men with less education and poorer backgrounds, as Max and Sam had, tended to see their inability to manage their health or recover from addiction as a moral failing–a self-accusation than led to shame and more addiction–and not as a more complex biological, psychological, social, or political problem. While having such a holistic understanding of their health status does not in and of itself lead to better outcomes, this critical consciousness has been shown to improve healthcare decision making and reduce shame, which I discuss elsewhere helped fuel relapses [20,35–37].

Interventions and treatment paradigms my informants experienced were rarely tailored to individual needs or took into account the complexities of strategic or tactical survival methods, let alone how these methods were tied to their identities, self-esteem, and life histories. All of the men had experienced large-scale, one-size-fits-all interventions like state-funded 30-day residential rehabilitation programs or court-mandated 12-step meetings; these were intensive or comprehensive. Of the men who had been incarcerated for drug-related offenses (N = 9), none received addiction treatment while in jail or prison or psychiatric treatment beyond medication. During my fieldwork, Sam was jailed for 100 days in the psychiatric ward of the San Diego County Jail. Despite having been a methamphetamine addict for three decades, HIV-positive for two decades, and diagnosed by other correctional physicians with various psychiatric disorders, he received no psychiatric treatment beyond the suggestion that he attend the weekly 12-step meeting.

Both Sam and William had at various times been part of the county’s intensive case management program, which combined substance abuse treatment, housing assistance, and employment assistance. It is based on the ACT model, which has been shown to be successful in a number of areas, included fewer hospital visits and improving overall quality of life [38,39]. A widely publicized ACT-based program focused on reducing the hospital costs of the 35 most expensive chronic homeless people in the San Diego County was successful in cutting their healthcare costs by half, but then the program found its own funding hard to sustain [40,41]. However, less expensive people are even lower budgetary priorities. As with many interventions shown in clinical trials to be effective, in the real world of budget cuts, undertrained staff, and agentive, resistant clients, intensive case management is much less effective. William claims to have helped himself, finding his case managers’ zero tolerance for drugs unhelpful, unrealistic, and annoying. In Sam’s case, one simple reason for intensive case management’s failure was that the case manager’s office, according to Sam, was not easy to get to on bus lines. But the case manager never left his office to come to see Sam and did very little to follow up with Sam if he did not make meetings or meet behavioral benchmarks.

Increased funding and improved provider training that could expand and bolster the reach of ACT programs like intensive case management and would certainly improve the likelihood that the health and quality of life of men like Sam would improve. However, in the current funding environment, and even with predicted shift of Drug War priorities from incarceration to treatment, ACT is likely to be considered too expensive and logistically complicated. Thus, there needs to be research into both new interventions and innovative policies that take into account the integrated biological, social, and political complexities of addiction, HIV, homelessness, and other syndemics.
